# Efficiently gap-filling reaction networks

**DOI:** 10.1186/1471-2105-15-225

**Published:** 2014-06-28

**Authors:** Mario Latendresse

**Affiliations:** 1 Bioinformatics Research Group/Artificial Intelligence Center, SRI International, 333 Ravenswood Ave, Menlo Park 94025, USA

**Keywords:** Flux Balance Analysis (FBA), Gap-filling, Systems biology, Reaction network, Linear Programming (LP), Mixed-Integer Linear Programming (MILP)

## Abstract

**Background:**

Flux Balance Analysis (FBA) is a genome-scale computational technique for modeling the steady-state fluxes of an organism’s reaction network. When the organism’s reaction network needs to be completed to obtain growth using FBA, without relying on the genome, the completion process is called *reaction gap-filling*. Currently, computational techniques used to gap-fill a reaction network compute the minimum set of reactions using Mixed-Integer Linear Programming (MILP). Depending on the number of candidate reactions used to complete the model, MILP can be computationally demanding.

**Results:**

We present a computational technique, called FastGapFilling, that efficiently completes a reaction network by using only Linear Programming, not MILP. FastGapFilling creates a linear program with all candidate reactions, an objective function based on their weighted fluxes, and a variable weight on the biomass reaction: no integer variable is used. A binary search is performed by modifying the weight applied to the flux of the biomass reaction, and solving each corresponding linear program, to try reducing the number of candidate reactions to add to the network to generate a working model. We show that this method has proved effective on a series of incomplete *E. coli* and yeast models with, in some cases, a three orders of magnitude execution speedup compared with MILP. We have implemented FastGapFilling in MetaFlux as part of Pathway Tools (version 17.5), which is freely available to academic users, and for a fee to commercial users. Download from: biocyc.org/download.shtml.

**Conclusions:**

The computational technique presented is very efficient allowing interactive completion of reaction networks of FBA models. Computational techniques based on MILP cannot offer such fast and interactive completion.

## Background

Flux Balance Analysis (FBA) is a method for analyzing genome-scale steady-state models of metabolic networks [1,2]. Constructing an FBA model often requires completing, or gap-filling, the reaction network. This completion is needed when the model does not grow under some specific growth condition (i.e., given sets of nutrients and secretions). *Gap-filling* is a computational technique to complete a reaction network based on FBA without referring to the genome. Indeed, a complete knowledge of the functionality of the genome provides a complete reaction network and no gap-filling would be needed. Essentially, given a set of candidate reactions, gap-filling suggests to add some of these reactions to the model so that the FBA model grows, but without guaranteeing that the enzymes for the added reactions exist in the organism. This computational approach to complete a network is viable if it is unknown how to complete the reaction network based on the genome.

To the best of our knowledge, all the current approaches to gap-fill [2-8] use Mixed-Integer Linear Programming (MILP) and compute a minimum set of reactions to add. But, searching for a minimum set of reactions using MILP is computationally expensive if the set of candidate reactions is large. For example, the MetaCyc [9] database contains more than 12,000 reactions. With such a set of candidate reactions, and searching for a minimum set of reactions, MILP typically takes from several minutes to hours, because the number of integer variables increases proportionally to the number of candidate reactions. Frequently, more than an hour is needed for optimally solving such MILPs. Section “Results” presents such concrete cases.

For another example, [8] reports that GapFind with GapFill took about 14 hours when gap-filling a specific FBA model of *C. neoformans*. GapFind/GapFill uses MILP to find metabolites that cannot be produced and gap-fill the network of reactions of the model to produce them.

Although MILP can find a minimum number of reactions, those reactions are still only plausible suggestions to complete a reaction network. The reactions still need to be analyzed to confirm their validity for the organism. This analysis can be done by searching the scientific literature for references to pathways based on the suggested reactions to add. FastGapFilling also needs such an analysis, but it also provides several sets of reactions from which a broader analysis can be done.

Moreover, these MILP computational techniques focus on finding the smallest set of candidate reactions to complete a model, but that set is not necessarily the most appropriate solution. For example, if only one reaction is needed to produce an essential compound, it might be the case that there is a solution with two reactions that better correspond to the taxonomy of the organism. Knowing the existence of this other non minimum solution might help discover the right missing reactions, which is a solution that might be given by FastGapFilling.

In this paper we present a computational technique using linear programming (LP), avoiding MILP, for reaction gap-filling. Solving a linear program with thousands and even tens of thousands of variables is typically about 1 second. Therefore, LP is a much faster computational technique, compared to MILP, that can give almost immediate feedback on what is needed to complete a reaction network. The gap-filling solutions found by FastGapFilling can be the same or different from those with the MILP approach, as the applications presented in Section “Results” demonstrate. In fact, several sets of candidate reactions are suggested by FastGapFilling, all offering positive growth condition, which may add more insight to complete a reaction network.

The next section presents the FastGapFilling algorithm based on LP.

## Methods

To the best of our knowledge, all computational techniques published to date for gap-filling a reaction network are based on MILP, including the one used by MetaFlux [6]. MetaFlux can solve FBA models, simulate knockout experiments, and gap-fill the reaction network, the secretions, the nutrients, and the list of biomass metabolites. In MetaFlux, the gap-filling process is based on *try-sets* and numerical value parameters, called weights, provided by the user. A try-set is a set of candidates to fill the incomplete model. For example, the try-set of reactions is a set, typically large, of candidate reactions for completing the reaction network. A try-set of nutrients would be a set of candidate compounds that could be transported into the organism to enable growth. Each type of candidates (reactions, nutrients, secretions, and biomass metabolites) has its own weight. For each candidate added to the model, its corresponding weight is added to a global objective function and MILP maximizes that objective. Therefore, a positive weight indicates a desire to include as many candidates as possible in the model, and a negative weight indicates a desire to include a minimum number. A typical scenario is assigning a large positive weight for the biomass metabolites and assigning relatively smaller negative weights to all other try-sets. Such a scenario includes as many as possible candidate biomass metabolites by adding a minimum number of reactions, secretions, and nutrients. In MILP, the inclusion/exclusion of each candidate is controlled by an integer variable taking only one of two values, 0 (do not include) or 1 (include). Thousands of such *binary variables* can exist because — besides the nutrients, secretions, and biomass metabolites — thousands of candidate reactions are typical.

**Figure 1:**
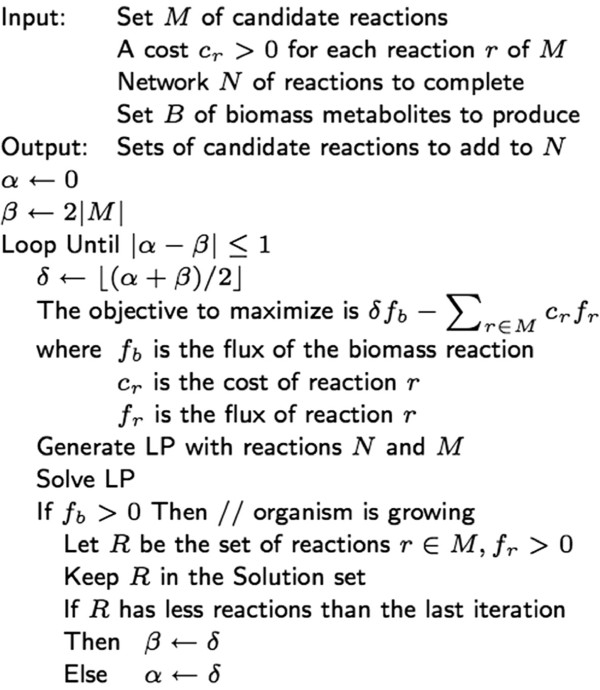
**Algorithm FastGapFilling to find sets of candidate reactions *R⊆M *to complete a network *N*.** A set *R* represents a set of candidate reactions suggested to be added to the model to achieve growth.

LP and MILP differ in one main respect: all variables in LP can take only fractional values, whereas some variables in MILP must have integer values (for example the binary variables). This difference alone makes MILP computationally more demanding. Indeed, a typical MILP solver must iteratively apply a series of LP problems to find the right integer values to satisfy all constraints. This process can be demanding when thousands or even hundred of thousands of LP cases need solving. In contrast, our technique typically requires solving only a few LP problems.The FastGapFilling algorithm is presented in Figure 1 and can be summarized in the following way: 

1. When creating the LP formulation, all candidate reactions *M* are included with the actual reactions *N* of the model to complete.

2. All biomass metabolites are combined as one biomass reaction as if the FBA model were solved. However, the objective function to maximize is the flux of the biomass reaction, multiplied by a weight, minus the sum of the fluxes of candidate reactions multiplied by the weights provided by the user (See Section “Results” for a discussion about these weights).

3. A binary search is performed based on the weight of the biomass reaction: the weight of the biomass flux is changed using a binary search between 0 and the number of candidate reactions. Each time the biomass reaction has a non-zero flux, the set of candidate reactions that are active is kept as one possible solution.

The algorithm does not try to gap-fill nutrients or secretions, because the model is assumed to have the correct growth environment specified before attempting to complete the reaction networks. Accordingly, our description of the algorithm does not discuss the growth environment.

The FastGapFilling algorithm accurately represents the model, because the entire biomass reaction is used as if solving the FBA model. The algorithm’s primary use is finding a small set of candidate reactions for adding to the model to produce greater biomass. In particular, if the biomass is zero (i.e., no growth is currently possible), the algorithm tries to obtain growth by activating candidate reactions. That particular case implements reaction gap-filling. Typically, *M* is a database of reactions such as MetaCyc, which includes approximately 12,000 reactions.

The generic reactions of MetaCyc are instantiated. That is, reactions that operate on classes of compounds are transformed to one or several reactions, where the classes are replaced by specific compounds that exist in the model. Any reversible reaction is transformed into two reactions, one for each direction, a standard practice in constructing FBA models. This transformation simplifies the mathematical modeling as the flux values of all reactions become non-negative.

The algorithm’s output is a list of reactions sets. An implementation may output only the smallest set that would suggest the reactions for model completion. But other implementations may present all sets found and let the user select the most biologically appropriate set. The algorithm does not necessarily find the smallest possible set of candidate reactions to complete the given model. It does try to reduce the size of that set, but it does not compute the absolute minimum set of candidate reactions to complete the model. In other words, FastGapFilling uses an heuristic searching for the smallest set of candidate reactions to complete a model, but it may not find the smallest set.

The search for a small set of candidate reactions to add to the model is based on one weight *δ* in the objective function. At each iteration of the search, that weight is adjusted either to help the solver increase the biomass flux *f*_*b*_ or to reduce the number of candidate reactions added. The number of iterations done is bounded by ⌈log2|*M*|⌉.

The costs *c*_*r*_ given as input to the algorithm are typically based on the kind (e.g., enzymatic vs. nonenzymatic) or taxonomic range (e.g., bacteria) of the reactions. The cost of a reaction is proportional to its probability of occurring in a model. For example, a reaction that is not known to occur in the taxonomic range of the model (e.g., a reaction only known in plants, but the model is for a bacteria) should have a higher cost than a reaction known to occur in the taxonomic range of the model. Examples of costs are presented in Section “Results”.

The mathematical formulation of the LP problem of this algorithm follows the standard approach of solving an FBA model, except for the objective function described below and specified in Figure 1. We refer the reader to the papers [2,4,6] that describe the generation of such LP. The statement “Generate LP with reactions *N* and *M*” in the algorithm assumes that sets of nutrients and secretions are given. Actually, the implementation guides the details of this generation, as it may include many other parameters, such as lower and upper bounds on the flux of some reactions, constraints to avoid ineffective high fluxes, and so on. Essentially, the generated LP must be solvable, must satisfy the steady-state constraints, and must restrict the fluxes to non-negative values for all reactions of *N* and *M*, including the biomass reaction.

The main novelty of the LP formulation is the objective function that changes at each iteration of the binary search. The objective function to maximize is $\delta f_{b}-\underset{r\in M}{\sum }c_{r}f_{r}$, where *δ* is a calculated weight by the binary search, *f*_*b*_ is the flux of the biomass reaction, and the summation of the terms *c*_*r*_*f*_*r*_ is done over all candidate reactions of *M*. This function tends to increase the flux of the biomass, as a maximization is applied and the *δf*_*b*_ is the only positive term in it. This function also tends to decrease the number of candidate reactions that have a non-zero flux (that is, these are active candidate reactions) because the term $-\underset{r\in M}{\sum }c_{r}f_{r}$ is negative (note that flux *f*_*r*_ and cost *c*_*r*_ are non-negative values). Indeed, this is the objective of the algorithm: increasing the flux of the biomass while decreasing the number of candidate reactions to add to do so. The essential point is to find the right value for *δ* such that *f*_*b*_ is non-zero (that is, growth is obtained, and the number of active candidate reactions is small). This is the objective of the binary search done by modifying *δ* such that growth is maintained and the number of active candidate reactions is reduced. Note that the initial value of *δ* is |*M*| (that is, the number of candidate reactions in *M*). This value enables many candidate reactions to be active simultaneously at the beginning of the binary search.

Note that, in general, this algorithm actually suggests adding candidate reactions to the model to increase the biomass. It could also be used for engineering more efficient metabolic pathways, although this aspect is not covered in this paper.

The main shortcoming of the algorithm is that it does not find a solution when no set of candidate reactions can be found to produce the entire set of biomass metabolites. In such cases, we recommend reducing the number of metabolites in the biomass reaction.

The following section demonstrates the application of FastGapFilling on an *E. coli* and yeast models.

## Results

We have applied the FastGapFilling algorithm to four incomplete *E. coli* models and to one incomplete yeast model. We started with a working *E. coli* model for which no added reaction are needed to obtain growth and then removed from 1 to 14 reactions in order to prohibit growth. A similar procedure was done to create the incomplete yeast model. See the following subsections for more details on the creation of these incomplete models.

The original *E. coli* model is based on the EcoCyc database (version 17.5). The model has 1,763 enzymatic reactions, including the instantiated generic enzymatic reactions. The biomass reaction has 69 metabolites. The growth media is composed of glucose, oxygen, ammonium, phosphate, diphosphate, sulfate, and iron. The secretions are carbon-dioxide, acetate, and water. An upper bound of 16 mmol/g/h was constraining the intake of glucose.

For each incomplete model, we also ran MetaFlux [6] in development mode to gap-fill the reaction network. The development mode of MetaFlux uses MILP with the SCIP [10] linear solver. The MILP gap-fill solutions were compared with the gap-fill solutions found by using the FastGapFilling algorithm.

In the MILP development mode, weights are user specified to control the candidate reactions to include in the model. They are called *costs* if their values are negative and *gains* if their values are positive. Costs are applied to reactions, and a gain, to the biomass metabolites. The gain is not user specified when using FastGapFilling, because the weight to the biomass reaction is controlled by the algorithm, but only for MILP. The following costs and gain were used for all cases presented in this paper. 

1. A cost of 1 for one candidate spontaneous (i.e., nonenzymatic) reaction

2. A cost of 5 for one candidate reaction inside the taxonomic range of the organism

3. A cost of 10 for one candidate reaction of unknown taxonomic range

4. A cost of 15 for one candidate reaction outside the taxonomic range of the organism

5. A gain of 500 for one candidate biomass metabolite

The relative values of these weights (costs and gain), as opposed to their absolute values, are important. For example, the cost of a spontaneous reaction is smaller than the cost of an enzymatic reaction, because a reaction that does not require an enzyme can occur in any organism. Similarly, the cost of a reaction that is outside the taxonomic range of the organism is set higher than the cost of a reaction in the taxonomic range of the organism because we prefer reactions in the taxonomic range of the organism selected. The candidate reactions are all coming from the MetaCyc database, and their taxonomic ranges are based on the curated pathways of that database.

Notice that the gain of one biomass metabolite is high compared to the cost of any reactions. For example, the ratio between the gain and the cost of a reaction in the taxonomic range of the organism is 100. This means that up to 100 such reactions could be added to the model if one more biomass metabolite can be produced. The intention is to produce a maximum number of biomass metabolites even if a large, but still bounded, number of candidate reactions is needed to produce them. As shown in the following subsections, this general result was obtained for all cases presented because a few reactions produced all metabolites.

Table 1 presents a summary of the results for the speed and number of reactions that the FastGapFilling algorithm and MILP suggested to be added. The following subsections present additional details about each application done.

**Table 1:** **Execution time of the FastGapFilling (FGF) algorithm compared with MILP on four incomplete models of *E. coli * and one incomplete model of yeast alongside the number of suggested reactions**

	**Time in seconds**		**Nb of reactions** **Suggested to be added**	
**Model**	**MILP**	**FGF**	**MILP**	**FGF**
*E. coli* 1	125	6	1	1
*E. coli* 2	7,794	16	3	3
*E. coli* 3	9,729	13	2	3
*E. coli* 4	>86,400	14	NA	3
Yeast	21,027	14	4	4

All times were rounded to the nearest number of seconds and they included only solver time, excluding the time for preparing the data for the solver. Note: the number of suggested reactions does not constitute an absolute measure of the quality of the solutions, but rather is one indicator of the similarity between the MILP technique and the FastGapFilling algorithm.

## Discussion

### First incomplete *E. coli* model

To create the first incomplete *E. coli* model, we removed the following reaction from the original *E. coli* model: 

Note: in this subsection and the following subsections, the MetaCyc reaction unique identifier is shown between parentheses. In the reaction just shown, that identifier is branched-chainaminotransferleu-rxn, which can be used as an unambiguous keyword to search for more information about that reaction at BioCyc.org.

That reaction, going right to left, is the last step in the biosynthesis pathway of the amino acid L-leucine and, going left to right, the first step in the L-leucine degradation pathway. No other reaction in the model produces L-leucine. Because L-leucine is part of the biomass reaction of the model, we expect that adding this reaction will be suggested when gap-filling is done. Indeed, the MetaFlux gap-filling MILP solution suggested adding the same reaction, in the right to left direction. MetaFlux could give the relevant direction as part of the solution because all reversible candidate reactions are split into two reactions, one for each direction. MetaFlux did not suggest adding the reaction in the opposite direction because the reaction was not essential for growth given the nutrients. SCIP required 125 seconds to obtain this optimal MILP solution.

When the FastGapFilling algorithm was run on the same incomplete model, adding the same reaction, in the same direction, was suggested. Four iterations (that is, four LP runs) were needed before that solution was found; the first three iterations found solutions with three reactions. The total solver running time of these four iterations was 6 seconds.

This first simple example shows that FastGapFilling can find the exact same solution as the MILP technique in much less time.

### Second incomplete *E. coli* model

The second incomplete *E. coli* model is derived by removing, additionally to the reaction removed in the first incomplete *E. coli* model, three reactions that produce the compound tetrahydrofolate, which is part of the biomass. These additional reactions are: 

Notice that the reaction of the previous incomplete model and these three reactions are in different metabolic pathways.

The MILP development mode of MetaFlux suggested adding three of the reactions that were removed: the first two reactions above, and the reaction removed in the first incomplete model. That is, the third removed reaction above was not suggested. The SCIP solver required 7,794 seconds or about 2:10 hours to find this optimal solution.

We ran the FastGapFilling algorithm on the same incomplete model. The smallest set of suggestd reactions included the same three reactions. Other solutions proposed included four reactions. FastGapFilling performed 12 LP solving iterations, with a solver total execution time of 16 seconds.

This second example shows that FastGapFilling can find the same solution as the MILP technique in substantially less time — in this case, well over 2 orders of magnitude faster.

### Third incomplete *E. coli* model

For this third example, we selected four reactions to remove from the original *E. coli* model tricarboxylic acid cycle (TCA cycle) pathway: 

The MILP solution suggested adding two reactions, both in the taxonomic range of *E. coli*: 

Although, these two reactions were not among the four reactions removed, the second reaction does produce the compound 2-oxoglutarate, one of the compounds produced by one of the reactions removed. Notice that the development mode of MetaFlux produces only one of the possible optimal solutions. Other different minimal cost solutions may provide the same value, but MetaFlux outputs only one of them. In this example, an optimal solution might include two of the four reactions removed, but we cannot confirm it. SCIP required 9,729 seconds or about 2:42 hours to find this solution.

However, FastGapFilling found a solution of three reactions after 12 iterations lasting 13 seconds, the first two reactions being the same as the MILP solution plus the following reaction: 

The third reaction appears to be redundant because its flux, as given by FastGapFilling, is 0.00056, whereas the fluxes of the other two reactions are the same at 0.110394. The flux of the third reaction is much lower than those of the first two reactions.

Indeed, the low-flux reactions suggested by FastGapFilling might simply be a way to increase the biomass and might not be reactions essential for growth. In general, this possibility can be verified by only adding the suggested reactions with relatively high fluxes and solving the resulting network. If the biomass can be generated, the low-flux reactions would be nonessential.

### Fourth incomplete *E. coli* model

The fourth incomplete model is the original *E. coli* model with 14 reactions removed. These reactions were selected because they produced at least one of the following metabolites: L-lysine, L-leucine, L-isoleucine, and L-histidine. All these metabolites participate in the biomass reaction. Essentially, this is an example where many biosynthesis pathways have been disturbed. The following reactions were removed. 

The SCIP solver could not find an optimal solution to the MILP problem after running for 24 hours. However, FastGapFilling produced the following solution, after 12 iterations that took 14 seconds by the SCIP solver, by suggesting three reactions: branched-chainaminotransferileu-rxn, diaminopimdecarb-rxn, and histaldehyd-rxn. These three reactions are among the 14 reactions that were removed.

This example shows that FastGapFilling can be very useful in practice: no optimal or near optimal solution could be found after 24 hours using the MILP approach, whereas FastGapFilling quickly found a gap-filling solution.

### Incomplete yeast model

As a last example of applying the FastGapFilling algorithm, we used a yeast model. As with the *E. coli* model, the original yeast model can grow. The original yeast model is based on the YeastCyc database (version 17.5). The model includes 1,454 enzymatic reactions, including the instantiated generic enzymatic reactions. The biomass reaction is composed of 41 metabolites. The growth media is composed of glucose, oxygen, ammonium, phosphate, sulfate, and iron. The secretions are carbon-dioxide, carbon-monoxide, formate, hydrogen-peroxide, glycolaldehyde, and water. An upper bound of 12 mmol/g/h was constraining the intake of glucose.

To generate an incomplete model, four reactions responsible for the biosynthesis of five lipids (ergosterol, zymosterol, episterol, fecosterol, and lanosterol) were removed. These five lipids are part of the biomass. The reactions removed are: 

The first reaction (GPPSYN-RXN) appears in three different pathways (trans-farnesyl diphosphate biosynthesis, geranyl diphosphate biosynthesis, and hexaprenyl diphosphate biosynthesis), whereas each of the other reactions occurs separately in three other pathways.

The optimal MILP solution for gap-filling this incomplete yeast model, required 21,027 seconds or about 5:50 hours, and suggested adding the four reactions above. This set is the least number of reactions expected because MetaCyc (version 17.5) includes no other reactions to produce these lipids.

FastGapFilling found the same solution, after 12 iterations lasting 14 seconds using the SCIP solver — a much faster execution time when compared to MILP. The binary search of FastGapFilling also found other solution sets with up to 34 candidate reactions, but the smallest set included the exact four reactions that were removed.

## Conclusions

FastGapFilling is an efficient technique to gap-fill reaction networks allowing interactive completion of reaction networks of FBA models, whereas MILP fails to offer such an efficiency.

The FastGapFilling algorithm increases the biomass flux by minimizing a weighted minimum sum of fluxes from new reactions, whereas the gap-filling approach using MILP is based on adding a minimum number of new reactions to obtain a non-zero biomass flux.

Using concrete examples, we show that the FastGapFilling algorithm is efficient for performing reaction gapfilling requiring much less computation time than MILP-based approaches. This result is not surprising because MILP may have to solve hundreds or even thousands of LP problems, whereas the FastGapFilling approach requires solving only a few LP problems.

## Competing interests

The author declares that he has no competing interests.
